# Threshold Concepts in Medical Education

**DOI:** 10.15694/mep.2018.0000176.1

**Published:** 2018-08-21

**Authors:** Virginia Randall, Robert Brooks, Agnes Montgomery, Lauren McNally

**Affiliations:** 1 Uniformed Services University of the Health Sciences

**Keywords:** Threshold Concepts, Medical student education, reflective practice

## Abstract

This article was migrated. The article was marked as recommended.

**Background** - The theory of Threshold Concepts (TC) proposes that there are ideas necessary for a student to learn which enable them to think like a professional. Studies of TC in higher education have appeared since 2003. Studies in medical education are more recent.

**Method** - We studied TC using a qualitative analysis approach (grounded theory and constant comparison) to produce a thematic analysis of 135 de-identified reflective practice essays from students in the pediatric clerkship at our medical school.

**Summary of results** - Seven themes met our criteria for a threshold concept; transformative (ontological shift) and troublesome (causes angst). 2 TC in our students’ work were identical with those found by authors from the UK (“Medicine isn’t black and white,” and “Sometimes there isn’t a right answer,”) 4 TC were similar and 2 were distinct.

**Discussion** - Our findings suggest that there are some TC inherent (maybe essential) in personal and professional identify formation for a student moving from layperson to physician-hood, regardless of the setting of the medical school.

## Introduction

Threshold concepts (TC) is a theoretical framework in higher education to describe ways of thinking and reasoning that are unique to a profession and enable the learner to “think like” and become a professional. The TC framework was developed by Meyer and Land ( Meyer and Land, 2003; Land, Meyer and Smith, 2008; Meyer, Land and Baille, 2010; Land, Meyer and Flanagan, 2016) who first published in 2003. The first TCs applied to economics and engineering, the disciplines of Meyer and Land. Since then, they have been studied in Europe, Australia, and New Zealand in such diverse professions as architecture, literature, social studies, and accounting. A handful of studies identifying TCs in medical education have appeared recently from authors in the United Kingdom ( Neve, Wearn and Collett, 2016a; Barradell and Peseta, 2017; Collett, Neve and Stephen, 2017; Neve, Lloyd and Collett, 2017b; Neve, Lloyd and Collett, 2017a).

There are four key components of a TC:

1. A TC is ***transformational***; it causes an ontological shift in the way the learner views himself or herself as a person/professional ( Mezirow, Taylor and associates, 2009). The learner comes to identify themselves in a new way, and appreciate their role in their new profession in a new way.
2. A TC is ***integrative***; it brings pieces of seemingly unrelated knowledge and attitudes into a whole, when the learner realizes, “Oh that is what they are talking about” “Now it all makes sense!” As faculty, we say “The light bulb went on!”
3. Intertwined with transformation and integration is ***irreversibility***; once the learner embraces the TC, he/she cannot unlearn it. It has become their professional identity.
4. A fourth component is ***troublesomeness***; a TC involves the angst that learners feel as they approach a new way of experiencing themselves and their role, coupled with their fear of this new unknown. We see medical students who want more involvement and responsibility for their patients, but who are simultaneously aware that their knowledge is not complete and are fearful of making a mistake. They oscillate between these two opposing ways of being and struggle to reconcile them. They are facing the uncertainty of medicine. During this troublesome phase, students may become anxious, depressed, cynical, and may contemplate dropping out of medical school.

Other descriptors of TCs are “boundedness”: the knowledge gained is specific to that discipline (in our study, to medical care). “Discursive” refers to the student using the language of the discipline, and fitting into the community of practice. Often times this may involve a period of mimicry, when the student is aware that they are using a “script” and has not yet transitioned to a natural use of this new language.

Collett and Neve and their colleagues in the UK ( Neve, Wearn and Collett, 2016b; Collett, Neve and Stephen, 2017; Neve, Lloyd and Collett, 2017a) have studied the TC encountered by medical students captured in audiodiaries, in which they found concepts including appreciating uncertainty, recognizing a bigger picture, not needing to know everything, and an appreciation of the physicians’ professional culture. Barradell and Peseta ( Barradell and Peseta, 2017) from Australia have provided a qualitative research synthesis of studies of TC in healthcare. They concluded that, taken as a whole, the studies from 2003-2014 included ideas that induct students into complex practice, enable them to work with new knowledge, and promote the development professional and personal agency.

TCs are important for faculty to appreciate, although remembering one’s own TC experiences is difficult for experienced faculty, since these concepts have become invisibly integrated into our own professional and personal identify ( Meyer and Land, 2006). As faculty, we can help students who may be stuck at a juncture with a TC by listening to their concerns, providing non-judgmental discussions, and most, of all, normalizing the experience for the student. That medical students suffer is undeniable, and we have all seen students who become depressed and decide to leave medical school in their clinical years. Ways of dealing with medical student suffering include: small group sessions, opportunities for protected venting, and guidance for reflection ( Egnew *et al.*, 2018).

We will examine TCs we identified in our pediatric clerks in the US and find a striking similarity with those identified in medical students in the UK, involving a different student population and a somewhat different method of analysis.

## Methods

We used the grounded theory and constant comparison approaches of qualitative analysis to generate a thematic analysis of reflective essays from our 3 ^rd^ year pediatric clerks. Reflective essays are a routine part of the curricular requirements at our medical school, and students write many during the course of the four years. The reflective essay in the pediatric clerkship is a requirement of the course and is ungraded. Clerks are informed in their student manual and by email of the assignment. Prompts are provided and the clerks are told that their essays will be de-identified and analyzed using qualitative analysis by a faculty member and one or more 4 ^th^ year students. Prompts for the written reflection are:

1. How have you changed since beginning your clerkship year?
2. What is the most important concept you have understood since beginning medical school that enables you to think like a physician?
3. What is the most difficult concept about being a physician you have encountered?
4. Describe any new approaches to life, medicine, or learning that you have developed this year.

Each clerk posts their essay on a confidential server, where it is downloaded by an administrator who removes all names and location references. Near the end of the clerkship, the clerks attend a facilitated group session where, if they wish, they can discuss their paper.

The essays were divided among three 4 ^th^ year medical students and a faculty member. Each pair of researchers followed the same process: independent line-by-line coding, then discussion between the pair of differences until resolution. There were 75 codes developed across the three sets of researchers. There followed independent thematic analysis within the pair, sorting the codes into themes and discussing the themes until there was complete agreement. At the end, there were 11 themes. It is worth noting that the 4 ^th^ year medical students showed great insight into the theory of TCs, and readily self-identified with many student struggles. They were able to explain the point of view of the 3 ^rd^ year clerks to the faculty member, and so created an understanding among the group.

The themes were analyzed to determine which ones rose to the level of a definition of a TC. We considered two characteristics to be essential: transformation and troublesomeness. Thus, within the many verbatim student quotes from their papers, we required evidence of a shift in personal identify and a struggle to achieve that shift.

The software programs HyperRESEARCH© and NVivo© were used to keep track of the quotes and codes. The medical students did their initial line-by-line coding using paper copy and colored pencils.

## Results/Analysis

Students generally wrote 1-2 pages. Many were descriptive of intensely emotional encounters with TCs. We collected 135 de-identified essays from the class of 2015. No student opted out. There were 75 codes and 11 themes. We found 1 core phenomenon code that represents the overarching theme for the data.

We found 7 TCs, which are illustrated with verbatim student quotes.

• “Being smart isn’t enough”
  • “The knowledge is the prerequisite, ***but it isn’t the thing itself.***”
  • “Caring for patients requires not only knowing the relevant knowledge, but ***also developing a human relationship with the patient.***”
• “It’s about the patient”
  • “Sometime since the first day, and I am not sure when, there ***came a point*** where I no longer cared about embarrassing myself or how I did things, but rather ***I started caring abut treating the patient*** and doing the best I could.”
  • “ ***that my needs***, whether it be hunger, thirst, sleep, a clean house or education, ***are always going to come second to my patient’s needs.***”
• “Life isn’t fair”
  • “ ***It taught me*** that not everything with youth has a happy ending - ***life isn’t always fair***.”
  • “This experience was ***difficult*** for me..This girl was supposed to begin her life, not have it end that day. It honestly ***took me a little while*** t get excited about medicine again.”
• “Sometimes there isn’t a right answer”
  • “ ***Not having the right answer*** the first time (or even at all) is ***very frustrating***.”
  • “The ***difficult balance*** between the standard of care and the patient’s autonomy.”
• “You can’t save everyone”
  • “I had to learn that ***you can’t and won’t save everyone***, even those who deserve it the most..this experience taught me that sometimes bad outcomes occur even when you do the best you can.”
• “Learning is lifelong”
  • “I find I am relearning everything I was taught with ***a new perspective.***”
  • “ ***Now when I study***, it’s because I want to, not because I have to.”
• “Medicine isn’t black and white, but almost always grey”
  • “I have ***come to understand*** that not many things in medicine are ‘by the book.’”
  • “The concept of ***not having the right answer*** the first time (or at all), ***is still very difficult***.”

The overarching theme of the threshold concepts is that ***“There is a disconnect between what I thought medicine was going to be and the reality.”*** This threshold concept was identified in this and every subsequent data set, and by each medical student co-investigator as the core of what they encountered during their clerkship year.

## Discussion

**Table 1.** Convergence of Threshold Concepts in Two Medical Schools

Collett et al. (2017) School of Medicine and Dentistry, Plymouth University, UK	Randall, et al (2017)Uniformed Services University of the Health Sciences, Bethesda, MD, US
Identical Ideas	
Medicine isn’t black and white but grey and complex.	Medicine isn’t black and white but almost always grey.
There is no single or morally correct answer.	Sometimes there isn’t a right answer.
Similar Ideas	
Being a doctor is more than just treating the symptoms	Being smart isn’t enough
Empathy. Two way conversation. Treating the whole patient.	It’s about the patient.
Idea Found Only in UK Analysis	
Being a doctor. Thinking like a doctor.	
Idea Found Only in Our Analysis	
	Medicine is a career of lifelong learning.
	You can’t save everyone.
	Life isn’t fair.

We read with great interest the papers of Collett and Neve and their associates in the UK, who, too, have studied TCs in medical education. Collett et al and Neve et al used audio diaries to collect students’ thoughts after a meaningful experience. They used qualitative thematic analysis to parse the TCs recorded in the diaries. The UK medical students are distinctly different from the students at USUHS: they are younger, have less experience in the health care sector, and are not in the military (as are the USUHS students.) Nonetheless, there was a striking similarity between the TCs that emerged from these studies; in fact, four are nearly identical. Table 1 demonstrates the convergence between the TCs described in the UK study and in our study. We did not initially analyze for boundedness, as did Collett. Neither study found evidence of irreversibility, probably because these studies occurred at a single time point.Taken together, these two studies suggest that there are a group of TCs that are important for medical students to grasp as they approach becoming a physician. The overarching concept confronting students is that the reality of medicine often doesn’t match their expectations.

## Conclusion

Our work and that of Collett and Neve illustrate the synthetic themes found by Barradell, especially that of working anew with knowledge in the health sciences (“Being smart isn’t enough”) and induction into the community of practice (“Medicine isn’t black and white”). These TCs may be universal to preparation for the responsibility of caring for patients. Alternately, these TCs may vary in medical school settings in societies very different from our own.

Our work is just beginning as we seek to understand more about TCs in medical education. We will be looking to partner with a medical school with a different culture, to determine which of these TCs are indeed universal in moving from student to physician-hood.

Limitations to our study include that it was conducted in one medical school, at one point in time. Some TCs might have been erroneously coded and not recognized as themes. As more medical education researchers study TC, new or reframed TC will emerge.

*Disclaimer*: The views expressed in this article are those of the authors and do not necessarily reflect the official policy or position of the Department of the Army, Department of Defense, nor the U.S. Government. Some authors are a military service member or a U.S. Government employee. This work was prepared as part of their official duties. Title 17 U.S.C. 105 provides that ‘Copyright protection under this title is not available for any work of the United States Government.’ Title 17 U.S.C. 101 defines a United States Government work as a work prepared by a military service member or employee of the United States Government as part of that person’s official duties. None of the authors have any financial or other conflicts to report.

## Take Home Messages

• Threshold concepts is a framework that proposes each profession has ideas that are necessary for students to understand that enable them to “think like” and become a professional. Threshold concepts involve an ontological shift in the learner, and often a struggle to fully integrate into their professional identity.
• Threshold concepts that may be universal in medical school include: “Being smart isn’t enough,” “Medicine isn’t balck and white,” and “Sometimes there isn’t a right answer.”
• Students may struggle as they begin io incorporate threshold concepts. Faculty can counsel them by listening, normalizing, and making the thresold concept explicit.

## Notes On Contributors

Virginia Randall, MD MPH, is Associate Professor of Pediatrics at the Uniformed Services University of the Health Sciences, Bethesda, MD. She served for 30 years as a pediatrician on active duty in the U.S. Army and has been at USUHS for 15 years. She always involves medical students as co-investigators in research.

Robert Brooks, MD is a Captain in the US Air Force, Class of 2018 at the Uniformed Services University of the Health Sciences. He graduated from Haverford College, PA with a BS in Biology and has been interested in epigenetic inheritance. He will continue his career as a pediatric resident at the Walter Reed National Military Medical Center, Bethesda, MD.

Agnes Montgomery, MD is a Captain in the US Army, Class of 2018 at the Uniformed Services University of the Health Sciences. She graduated from Harvard with a BA in French literature and has been active in varsity tennis and the paraolympics. She will continue her career as a pediatric resident at the Tripler Army Medical Center, Honolulu, HI.

Lauren McNally is a 2LT in the US Army, Class of 2019 at the Uniformed Services University of the Health Sciences. She graduated from the University of Virginia with a BS in Education (Communication Disorders). She has been active in health care delivery to homeless and rural Americans.
