# Supplementary material for: Threshold Concepts in Medical Education
Source: MedEdPublish (2016). 2018 Aug 21;7:176. [Version 1] doi: 10.15694/mep.2018.0000176.1 (PMC10701810; doi:10.15694/mep.2018.0000176.1)
Supplement: Peer review history

# Reviewer report: Reviewer response for version 1

**DOI:** 10.21956/mep.19643.r29275

**Published:** 2018-08-26

**Authors:** Faraz Khurshid

**Affiliations:** 1 University of Buraimi, College of Health Sciences

This review has been migrated. The reviewer awarded 4 stars out of 5 Dear Dr Randall & Team. My humble gratitude and regards for sharing this interesting chunk of information. My interest lies in Threshold concept; though I often find my ideas oscillating in the liminal space, the intriguing quality of the threshold concept keeps my curiosity still breathing. Threshold concepts can be supported by Mezirow’s theory of Transformative learning in addition to the grounded theory. I liked the methodology of using Reflective essays with triggers/prompt to discern the threshold concept. In fact, the comparison and convergence of findings of two medical schools across two continents is a remarkable observation. As you mentioned that few of TC may exist as the inherent ideas in personal and professional attribute formation of a student. Acknowledging these concepts will offer valuable attributes in an individual transformation as a professional. Medical care and professionalism come under the umbrella of the threshold concept. The work done by you and your team is significant in addressing these concepts during pre-clinical and clinical years. Though, there may exist an imperative need to recognize the threshold concepts that may put a novice in liminality at the entrance level of a medical school. I am wondering if you and your team have any 'Big idea' of discerning the Threshold concepts that students might come across during the first two years of the medical school? Good work ��

Reviewer Expertise:

NA

No decision status is available

# Reviewer report: Reviewer response for version 1

**DOI:** 10.21956/mep.19643.r29277

**Published:** 2018-08-23

**Authors:** Julie Browne

**Affiliations:** 1 Cardiff University School of Medicine

This review has been migrated. The reviewer awarded 3 stars out of 5 This was a really enjoyable and interesting read and the description of Threshold Concepts (TC) will be valuable to anyone who has not encountered this theoretical framework before. It involved the coding and analysis of 135 reflective essays – a substantial piece of work. I particularly liked the practical messages for medical educators who will be supporting students as they tackle this ‘troublesome’ learning.My only concern is that, even though the results of the work appear to align well with previous studies, the authors indicate that they used only two of Meyer and Land’s original criteria to identify threshold concepts in their written data. No reason is given for this decision to modify the framework, and they don’t report whether (or how far) they also took into account the integrative, irreversible, bounded and discursive components that characterise a true threshold concept (rather than just an important insight). The integrative component (the 'light-bulb' or 'aha!' moment) is particularly important to the idea of the threshold concept because without it, students will struggle to scaffold and assimilate new knowledge in the future. It is therefore not clear that what the authors actually report can truly be classified as threshold concepts, even though the results certainly appear persuasive.

Reviewer Expertise:

NA

No decision status is available

# Reviewer report: Reviewer response for version 1

**DOI:** 10.21956/mep.19643.r29274

**Published:** 2018-08-23

**Authors:** Flora Smyth Zahra

**Affiliations:** 1 Faculty of Dentistry, Oral &Craniofacial Sciences. King's College London

This review has been migrated. The reviewer awarded 3 stars out of 5 It is always interesting to consider aspects of MedEd as a series of thresholds. I do however feel the article would have been strengthened by mention of some of the work already carried out in areas closer to Medicine than just the original Land and Meyer work. If you haven't come across it before it might interest the authors to look at work carried out in Dentistry - As they say in the article below TC's offer, ''a role for the student voice in offering a novice perspective which is paradoxically something that is out of reach of the subject expert. Finally, the application of threshold concepts highlights some of the weaknesses in the competency-based training model of clinical teaching.'Threshold concepts in dental educationhttps://www.ncbi.nlm.nih.gov/pubmed/21985204

Reviewer Expertise:

NA

No decision status is available

# Reviewer report: Reviewer response for version 1

**DOI:** 10.21956/mep.19643.r29278

**Published:** 2018-08-22

**Authors:** Tan Nguyen

**Affiliations:** 1 Deakin University

This review has been migrated. The reviewer awarded 4 stars out of 5 This paper provide insight into the work on Threshold Concepts and is highly relevant to medical education. Whilst the discussion from the literature is a little light in this space, I suspect it could from limited published work in the area. It would be interesting to see how and when the 'irreversibility' component (an area for potential to investigate) is evaluated around professionalism and/or professional identity, because this would have implications for how clinical educators can assist student learning throughout their course program. How 'soft skills' are taught is more difficult to evaluate, unlike the development of clinical skills, which can be more objectively assessed.

Reviewer Expertise:

NA

No decision status is available

# Reviewer report: Reviewer response for version 1

**DOI:** 10.21956/mep.19643.r29276

**Published:** 2018-08-21

**Authors:** Trevor Gibbs

**Affiliations:** 1 AMEE

This review has been migrated. The reviewer awarded 4 stars out of 5 I very much enjoyed reading this paper and understanding threshold concepts- I feel that the paper's importance lies with the idea rather than its congruence between schools; however I think it does show how schools think. I thought that the references were particularly helpful and I would suggest that this paper is a good read for all involved in curriculum planning, although I do believe that implanting these threshold concepts will not be a simple process.

Reviewer Expertise:

NA

No decision status is available

# Reviewer report: Reviewer response for version 1

**DOI:** 10.21956/mep.19643.r29273

**Published:** 2018-08-21

**Authors:** Adina Kalet

**Affiliations:** 1 New York University School of Medicine

This review has been migrated. The reviewer awarded 4 stars out of 5 This paper is a wonderful example of the value of the MedEdPublish pre-peer review publishing format! The title was clear and informative, the paper summarized a new idea in medical education very briefly and the paper itself was brief, well written, put their work in context of the literature and provided preliminary research findings that illustrates the value of this theory to our community. This is a good conversation starter and I will be sending this link to my MHPE students and tweeting this to get the word out, I also found the Threshold Theory itself interesting (thanks for introducing me to Neve, Meyer and Land) and important to my own work in Professional Identity Formation and relevant to those interested in exploring the integration of basic science concepts into clinical training. Good work, I look forward to to his ongoing conversation in the literature.

Reviewer Expertise:

NA

No decision status is available
